# How Did Host Domestication Modify Life History Traits of Its Pathogens?

**DOI:** 10.1371/journal.pone.0122909

**Published:** 2015-06-19

**Authors:** Marie De Gracia, Mathilde Cascales, Pascale Expert, Marie-Noelle Bellanger, Bruno Le Cam, Christophe Lemaire

**Affiliations:** 1 INRA, IRHS, SFR QUASAV, Beaucouzé, 49071, France; 2 Université d’Angers, IRHS, PRES UNAM, SFR QUASAV, Angers, 49045, France; 3 Agrocampus Ouest, IRHS, SFR QUASAV, Angers, 49045, France; University of Nebraska-Lincoln, UNITED STATES

## Abstract

Understanding evolutionary dynamics of pathogens during domestication of their hosts and rise of agro-ecosystems is essential for durable disease management. Here, we investigated changes in life-history traits of the fungal pathogen *Venturia inaequalis* during domestication of the apple. Life traits linked to fungal dispersal were compared between 60 strains that were sampled in domestic and wild habitats in Kazakhstan, the center of origin of both host and pathogen. Our two main findings are that transition from wild to agro-ecosystems was associated with an increase of both spore size and sporulation capacity; and that distribution of quantitative traits of the domestic population mostly overlapped with those of the wild population. Our results suggest that apple domestication had a considerable impact on fungal characters linked to its dispersal through selection from standing phenotypic diversity. We showed that pestification of *V*. *inaequalis* in orchards led to an enhanced allocation in colonization ability from standing variation in the wild area. This study emphasizes the potential threat that pathogenic fungal populations living in wild environments represent for durability of resistance in agro-ecosystems.

## Introduction

While the impact of domestication on many organisms has been well described [1–4], little attention has been paid to the evolutionary dynamics of pathogens during domestication of their hosts and rise of agro-ecosystems [5, 6]. Understanding how life history traits of pathogens might have been affected through the crucial process of domestication of their host is particularly relevant. Domestication of plants and animals is one of the most important advances in agriculture in the past 13,000 years. It can be defined as an evolutionary process triggered by the anthropogenic selection of individuals from wild populations based on agronomic criteria [7]. Continuous selection of wild species to new anthropic environments like agro-ecosystems has governed our current diet, the rise of civilizations and the world population demography. Anthropic selection based on some phenotypic traits resulted in a strong accumulation of fixation of alleles, some of them being adaptive, in the genomes of domesticated plants [1, 4, 8], animals [9] but also microorganisms [10]. The set of morphological and/or genetic differences between domesticated species and their wild progenitors defines the domestication syndrome [11, 12]. Among pathogens attacking our basic food resources, fungi cause more than a third of emerging diseases [13, 14]. The question here is how much does host domestication change the adaptive landscape for pathogens? Understanding how pathogens living on wild hosts adapt to agricultural constraints would allow us to manage emerging diseases and improve the sustainability of host genetic resistances.

Beside the proper phenotypic evolution of the plant during domestication, the influence of transition from natural to domestic environments on host-pathogen co-evolution is not negligible. Phenotypic values of plants living in agro-ecosystems are expected to be quite different from those measured in the wild. Indeed, natural environments are characterized by a random distribution of host plants, low plant density, high genetic diversity and uneven distribution of nutrients [6]. In contrast, the development of agriculture favored the ability of plants to grow in new habitats with higher densities, but less genetic diversity, increased and uniform nutrient availability and less interspecific competition. The ability of a pathogen to adapt to this new environment therefore depends on its life history and potential for evolution of traits [14, 15]. Recently, the term pestification was introduced for the first time by Saleh *et al*. [16] to describe the influence of rice domestication on its associated fungal pathogen *Magnaporthe oryzae*. In this study, the authors revealed modifications in the genetic structure of the pathogen’s population during its evolutionary history without any investigation on life history traits. Here, we define the term pestification as the process by which a pathogen originally living in the wild become adapted to biotic and abiotic crop conditions. Among traits affected by pestification, two categories are particularly relevant in epidemiology: growth and dispersal [17]. Intrinsic hyphal growth is a classical measurement of fitness [17, 18]. Fungal pathogens are often characterized by a dispersive phase mediated by asexual (mitotic) spores also called conidia. Criteria like the number of conidia produced, their size, and their germination rate are good predictors of fitness [17] and are relevant to determine the ability for dispersal. Determining how growth and dispersal of fungal pathogens have evolved during domestication of their host would help us better understanding their adaptive dynamics.

The pathosystem *Malus spp*.*-Venturia inaequalis* is particularly relevant to investigate changes in life history traits of pathogens that are adapted to domesticated hosts and agro-ecosystems. Indeed, the life history of *V*. *inaequalis* confers to this fungus a significant potential for evolution [15]. *V*. *inaequalis* produces both ascospores, spores issued from sexual reproduction, and conidia. Intrinsic dispersal ability of both types of spores is low, the most favorable way for the pathogen to achieve long distance dispersal being linked to human activities, such as transport of infected fruits and plants [19, 20]. It is assumed that the population structure of a pathogen is linked to the history of the spread of its host [21, 22]. Cultivated apple was domesticated in Central Asia from *Malus sieversii* [23–25]. This species makes large forests from the Tien Shan mountains (border of Kazakhstan, Kirghizstan and Xinjiang Province in China) to the Caspian Sea [26, 27]. Migration westwards have involved contact and hybridization with close wild relatives growing along the Silk Road, including *M*. *sylvestris* in Europe and *M*. *orientalis* in Caucasus [28]. These wild apple species have thus also contributed to the genetic mixing of domesticated apple. Previous genetic studies on *V*. *inaequalis* have provided many clues about its origin. A global analysis of diversity showed that *V*. *inaequalis* shares a common origin with its host in Central Asia [22]. Impact of apple domestication on genetic structure of the pathogen has then been investigated [29]. Focusing their study in Central Asia, the authors identified a population sampled on *M*. *sieversii* in natural forests of Kazakhstan, which was considered to be a relic of the ancestral population from which have been derived populations currently present in agro-ecosystems worldwide. More recently, a study focusing on pathogenicity traits of this ancestral population showed that apple domestication had markedly modified pathogenicity of *V*. *inaequalis* [30]. Indeed, the domestication process was associated with acquisition of virulence and increase of aggressiveness of *V*. *inaequalis*. The availability of valuable genetic resources and knowledge on this pathosystem provide an excellent framework to investigate the impact of host domestication on evolution of pathogen life history traits.

Here, we compared several life history traits of *V*. *inaequalis* isolates that were sampled in the ancestral and domestic populations in Kazakhstan. We chose traits that are known to be involved in dispersal and colonization success of fungal pathogens: mycelium growth, asexual spore germination rate, number of asexual spores and size of asexual spores. We addressed the following questions: (*i*) Are strains sampled in the domestic habitat phenotypically different from those isolated in the wild habitat? If so, which traits are involved in these differences? (*ii*) Did domestication of apple trees drive evolution of *V*. *inaequalis* to a new adaptive peak rather than to a standing phenotypic combination selected for life into agroecosystems? (*iii*) What are the implications of an eventual phenotypic change on the life history of *V*. *inaequalis* in agro-ecosystems?

## Material and Methods

We state that no permit was necessary to sample apple tree leaves containing the fungus *Venturia inaequalis* in both wild and agricultural locations in Kazakhstan. The field study did not involve endangered nor protected species.

### Fungal isolates

This study was based on a total of 60 isolates of *Venturia inaequalis* sampled on the wild apple *M*. *sieversii* in Kazakhstan. Thirty were collected in the wild forest area (43.23°N, 77.28°E) and 30 in the suburbs or agricultural area around Almaty (43.25°N, 76.91°E), the former capital of Kazakhstan. All these isolates were collected in 2006. Throughout the study, the terms wild population (CAM for Central Asian Mountains) and domestic population (CAP, Central Asian Plains) are used to make reference to a collection of individuals sampled in non-anthropic area and in anthropic area, respectively, as defined in [29].

### Phenotypic traits measurements

#### Hyphal growth

Estimation of the mycelium growth capacity of isolates was performed on malt agar medium. For each isolate, eight plugs of mycelium from a two-week-old culture were grown on a malt agar medium. Cultures were kept at 18°C. Considering that *V*. *inaequali*s growth is circular, we measured colony diameters using a ruler 14 days after mycelium plugs deposit. Two measurements were performed for each plug, which represents 16 measurements per isolate.

#### Spore traits measurements

Spore traits analysis included measuring spore germination rate, sporulation capacity and spore size. Initial inocula were obtained by growing single spores of each isolate on cellophane sheets deposited onto malt agar medium.

Germination rates were assessed for each isolate on malt agar plates covered with a cellophane sheet. Plates were kept in darkness at 18°C to allow conidia germination. After 24 hours, germinated and non-germinated spores were counted using a microscope. The *in vitro* germination rate was then defined as the percentage of germinated spores after 24 hours. For each isolate, a single germination rate was calculated.

Concerning sporulation capacity and spore size measurements, 10 μL of the initial inoculum was calibrated to 105 conidia.mL-1 and deposited on four malt agar Petri dishes covered by cellophane sheets of 3 cm2. Petri dishes were then incubated for seven days at 18°C under the light. After this period, cellophane sheets were water rinsed for measurements of spore size and spore concentration using a spore counter (Counter Multisizer, Beckman). Sixteen measurements were made for each isolate. The counter was calibrated to count only particles with a diameter in the range from 6.5 to 12.5 μm (range size of *Venturia inaequalis* conidia).

### Statistical analyses of the phenotypic data

All statistical analyses were performed using R Version 3.0.0 [31]. In order to test for independence between life history traits and for any trade-off change within each population, we performed pairwise Pearson's correlation tests. Pearson's correlation coefficients were estimated using the “corrgram” package [32].

For each life history trait, distributions were checked for normality using the Kolmogorov-Smirnov test. To test for differences between populations in each phenotypic variable, we used different procedures according to the availability of replicates. For differences between populations in sporulation capacity and spore size, an analysis of variance (ANOVA) was performed with a linear mixed-effect model (LME) implemented in the “nlme” package [33]. The « individual » factor was treated as random effect and nested in « population ». Germination rate for each isolate were analyzed using Student’s tests. Percentages were transformed by a logit transformation. Population effect on variation in hyphal growth was analyzed using an ANOVA. In all ANOVAs, normality of residuals was checked.

## Results

### Quantitative distribution of life history trait variables

**Fig 1:**
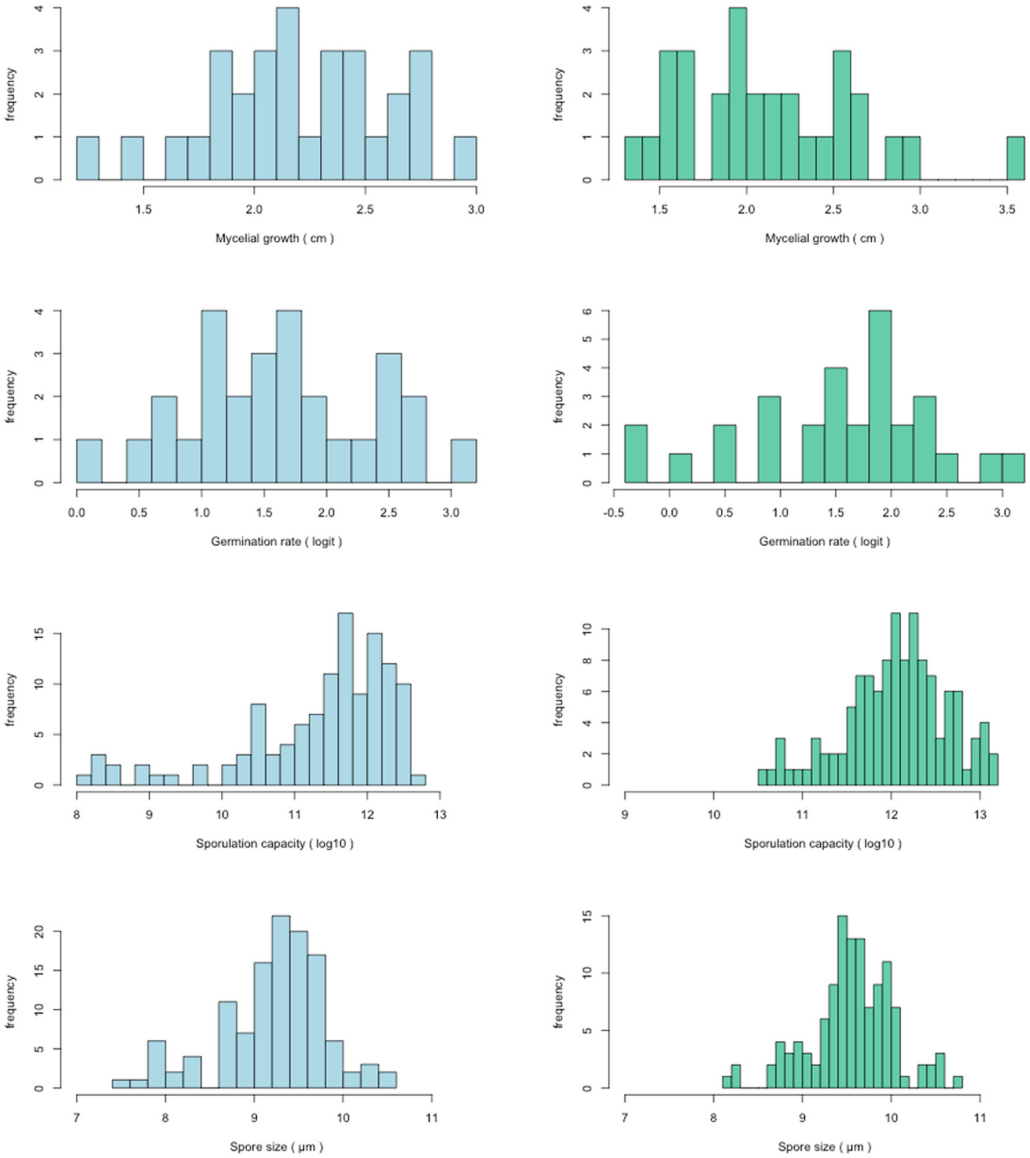
Quantitative distribution of life history trait variables. Distribution of quantitative variables: hyphal growth 24 days after deposits on agar (cm), germination rate (in logit units), sporulation capacity (in Log_10_ units), and spore size (μm). Blue histograms correspond to the wild population and green ones correspond to the domestic population.

The distribution of each trait measurement is presented in Fig 1. Analyses of hyphal growth were based on diameter measurements 24 days after mycelium plugs were deposited. The overall hyphal growth was between 1.29 and 3.53 cm in diameter. Data ranged from 1.28 to 2.94 cm (mean ± SD: 2.22 ± 0.41) in the wild population and from 1.39 to 3.53 cm (mean ± SD: 2.14± 0.50) in the domestic population. Spore analyses compared spore germination rates, sporulation capacity and spore size.

For the overall sample, germination rate varied between 45 and 96%: this rate ranged from 54 to 96% (mean ± SD: 81 ± 10.82) in the wild population, and from 45 to 96% (mean ± SD: 79.6 ± 14.08) in the domestic one. Sporulation capacity fluctuated much more than the other characters. The lowest sporulation capacity (3,250 spores mL^-1^) was observed in a strain from the wild population and the highest in a strain originating from the domestic population (534,400 spores mL^-1^).

Distribution for each population ranged from 3,200 to 298,300 spores mL^-1^ (mean ± SD: 122,400 ± 80,000) and from 39,600 to 534,400 spores mL^-1^ (mean ± SD: 200,000 ± 108,100) for wild and domestic populations, respectively. Concerning the spore size, isolates from the wild population ranged from 8.12 to 10.15 μm (mean ± SD: 9.26 ± 0.44), while those from domestic isolates ranged from 8.45 to 10.45 μm (mean ± SD: 9.55 ± 0.36).

### Correlation between life history traits

**Fig 2:**
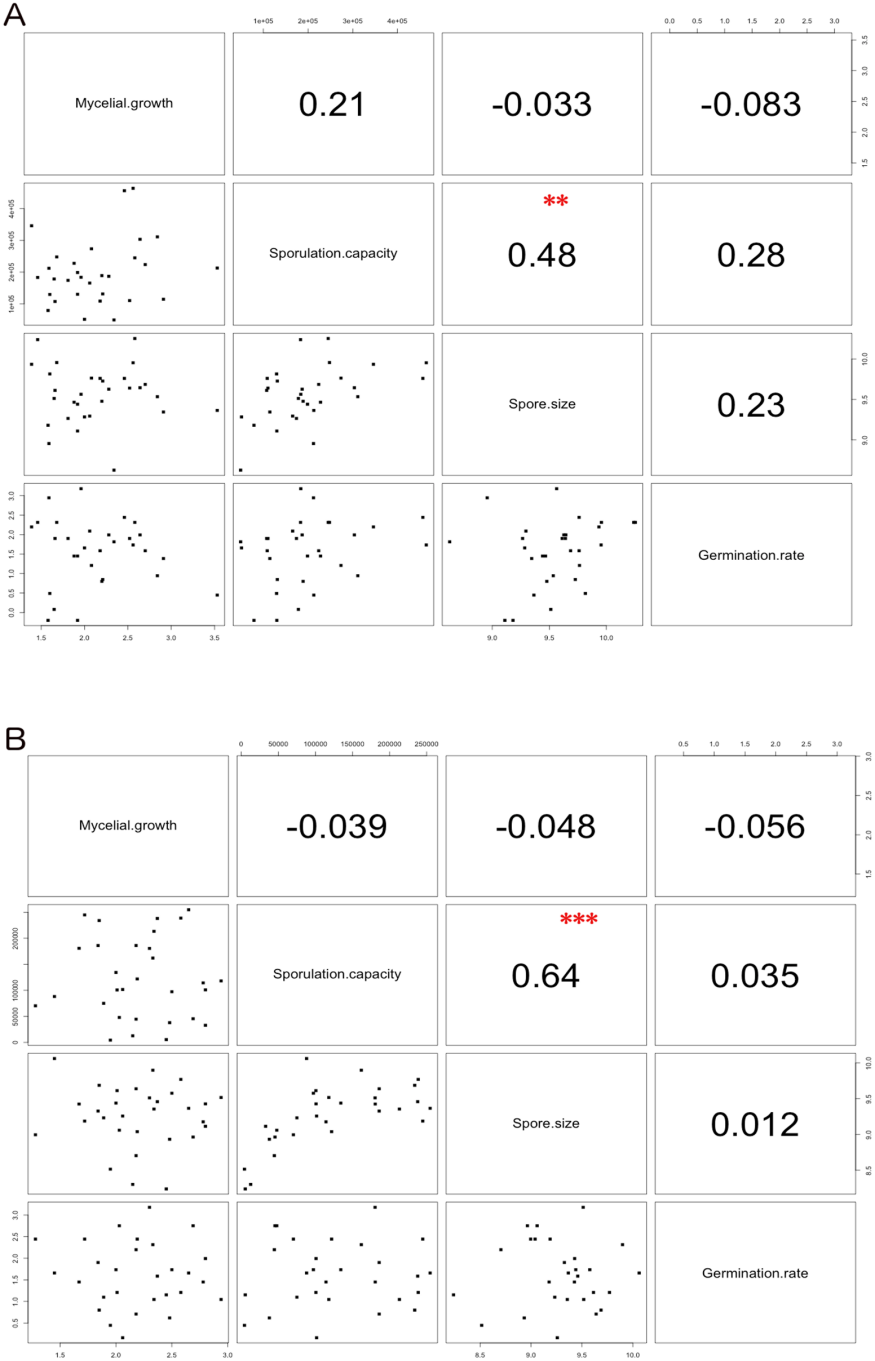
Pairwise correlations between phenotypic traits. Scatter plots indicating the degree of correlation between traits (below diagonal) and pairwise Pearson's correlation R values (above diagonal). Pearson’s correlation coefficients were calculated from averaged data from each strain for sporulation capacity, spore size and hyphal growth. Statistical significant correlation coefficients are indicated as following: ** P-value < 0.01 and *** P-value < 0.001. Calculations were performed for domestic (A panel) and wild (B panel) populations, respectively.

Pearson's correlations were tested between life history traits and pairwise correlation analyses were performed. Spore size was significantly correlated with sporulation capacity in each population (Fig 2). No other correlation with hyphal growth or germination rate was detected. The germination rate of conidia was not correlated with any other trait, meaning that the ability to germinate does not determine the size of the spores, nor the sporulation capacity.

Spore size was significantly correlated with sporulation capacity, with Pearson's correlation coefficients of *R =* 0.64 in the wild population (*P<0*.*001*) and *R =* 0.48 in the domestic pone (*P<0*.*01*) (Fig 2).

### Life traits differences between wild and domestic populations

**Fig 3:**
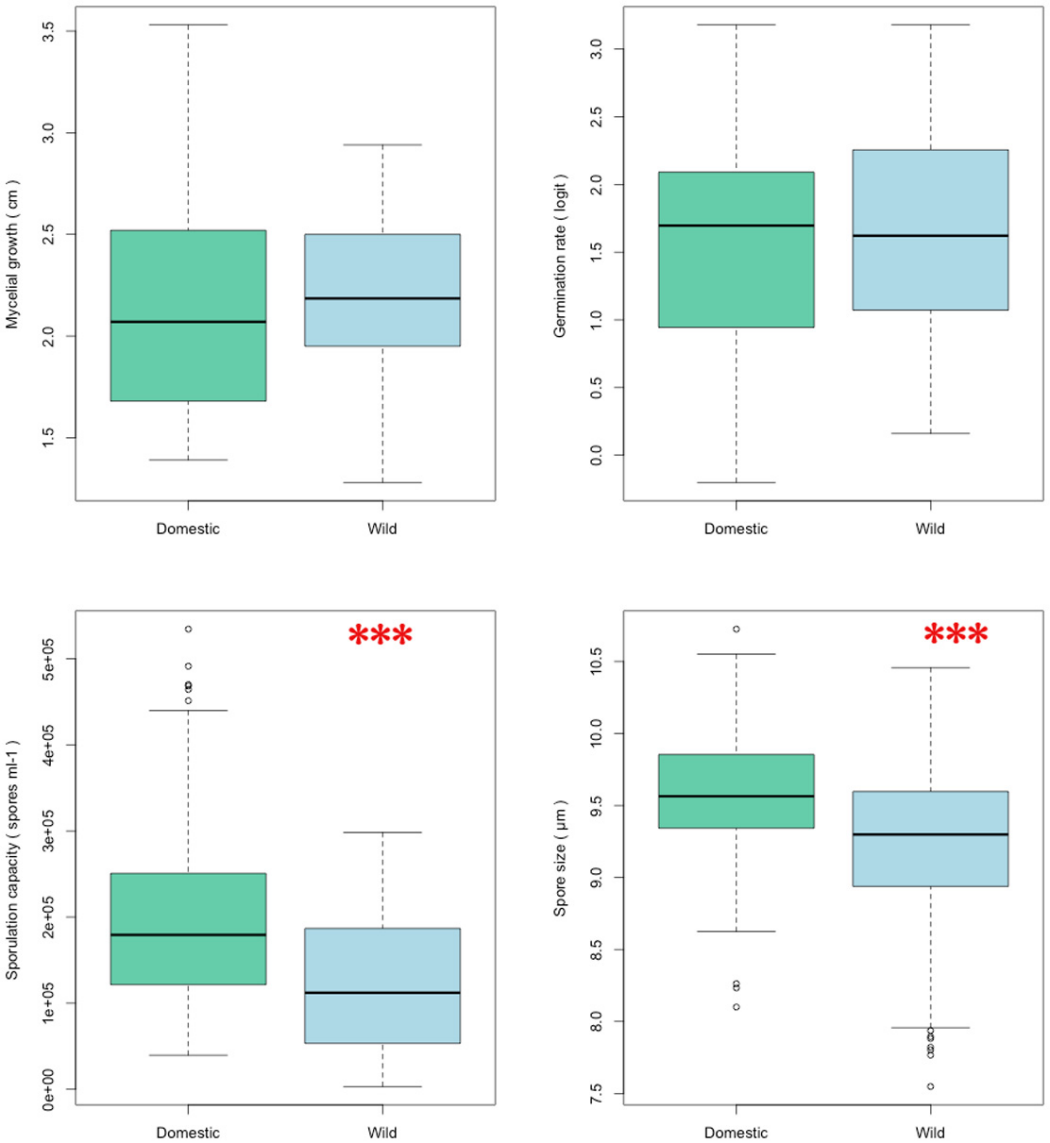
Quantitative distribution of life history trait variables between wild (blue) and domestic population (green). The horizontal line in boxplots represents the median. A significant difference between populations is observed for the sporulation capacity and spore size. Sporulation average in wild population: 122,400 spores mL^-1^; and in domestic population: 199,955 spores mL^-1^. Spore size average is 9.2 μm in the wild population and is 9.5 μm in the domestic population.

The germination rate of the wild population was not significantly different from the rate of the domestic one (81% and 79.6%, respectively) *(Student test; t = 0*.*29; P = 0*.*77)* (Fig 3).

Hyphal growth of the wild population was not significantly different from that one of the domestic population. Twenty-four days after deposition, average hyphal growth was 2.21 and 2.13 for the wild and domestic populations, respectively *(ANOVA*, *F = 0*.*40; P = 0*.*52)* (Fig 3).

A significant difference in sporulation capacity between the two populations was detected *(ANOVA; F = 188; P<0*.*0001)*. The domestic population exhibited a sporulation rate higher than that of the wild population. The average rates in the domestic and wild populations were 200,000 spores mL^-1^ and 122,400 spores mL^-1^, respectively (Fig 3). The spore size of isolates from the wild population was significantly smaller than the spore size of isolates from the domestic population (*ANOVA; F = 19085; P<0*.*0001*). Average size of the domestic population was 9.5 μm and that one of the wild population was 9.2 μm (Fig 3). For these two phenotypic analyses, we noted that distributions of both populations largely overlapped, with nearly all values of the wild isolates encompassed by the distribution of values of the domestic isolates. This result suggests that isolates sampled in the anthropic area were actually a sampling from the largest and most sporulating isolates of the wild population (Fig 3).

Our results demonstrate that apple domestication promoted modifications in traits related to dispersal of the apple scab fungus, *Venturia inaequalis*. Among the four life history traits that were analyzed, both spore production and size showed significant difference between the two tested sampling sites. Our results show that diversity in spore size and production is significantly higher in wild habitats than in agricultural ones.

## Discussion

Impact of plant domestication on pathogenic organisms has rarely been investigated despite its major importance in understanding consequences of modern human activities on disease emergence. Previous identification of fungal genetic resources that did not experience agriculture among all other human activities allowed us addressing this issue of great interest in evolutionary ecology. We used several phenotypic comparisons to investigate changes of different life history traits involved in dispersal of the apple scab fungus, associated with the domestication of its host. We chose strains that had been sampled on *M*. *sieversii*, the main wild Asian ancestor of the domestic apple tree, located in a wild forest and in the suburbs of Almaty. As previously mentioned, strains from the latter location, although sampled on *M*. *sieversii* trees, have been shown to belong to a population that is also present on domestic apple trees [29, 30]. Those strains were probably imported with domestic apple in Kazakhstan in the late XIX^th^ century. Indeed, because Kazakhstan was primarily inhabited by nomads, agriculture was not developed in this area before the annexation by the Russian Empire and colonization by Russian settlers. Selecting strains living on the same host species prevents from confounding effects due to adaptation to the host genotype. Our two main findings are that (1) transition from wild to agro-ecosystem was associated with an increase of the average of both spore size and sporulation capacity; and (2) that distribution of each quantitative trait of the domestic population mostly overlapped with those of the wild population. Our results suggest that transition from wild to orchard had a considerable impact on fungal characters linked to its potential for dispersal by selection from standing phenotypic di*v*ersity. In the present study, we showed that pestification of *V*. *inaequalis* in orchards led to an enhanced allocation in colonization ability for which potential capacity did already exist in the wild area.

### Correlation between fitness traits

The analysis of fitness-related traits is complex because fitness is a synthetic function of both survival and reproductive success. Disentangling between these two fitness components implies the measurement of several traits and the analysis of their pairwise correlations.

Our results clearly showed that spore production and spore size on the one hand were not correlated with mycelial growth on the other hand (Fig 2). A likely explanation would be that growth of *Venturia inaequalis* is independent of its ability to produce asexual spores. In other words, the quantity of mycelium produced per time unit does not impact the net production of conidia. This result is at odds with observations made on *Aspergillus niger* for which the mycelium area was significantly correlated with spore number [34]. Mycelium of *Venturia inaequalis* grows under the cuticle of the leaf and is therefore not visible. Aggressiveness is usually determined by the size of lesions harboring conidiospores on the leaf surface. Our results suggest that this way of measuring aggressiveness might not reflect the growth of *Venturia inaequalis* within the leaf tissues. Nevertheless, our results were obtained *in vitro* and need to be validated *in planta*, for instance using thermography or fluorescence-based methods [35]. In addition in our study, hyphal growth was analyzed from measurements of the diameter of the mycelium surface of each isolate. Further analyses would be useful because a mycelium is a network formed by a succession of hyphae with similar branching patterns at different scales that fit fractal geometry [36, 37]. A description and modeling of the fractal geometry of mycelium may be more appropriate to evaluate mycelium growth. Spore size is known to be a trait related to dispersal abilities of fungi. Indeed, smaller fungal spores are currently considered as fitter than large spores because they are more effectively dispersed [17]. Modeling the dynamics of airborne propagules clearly showed that spore size is negatively correlated with dispersal ability [38, 39]. However, in their recent theoretical study that looked at particle size ranging from 1 to 10 μm (the actual size of conidia in our study), Norros *et al*. [40] claimed that "*the dispersiveness of especially the smallest spores is so high that landing may in fact be a bigger challenge for them than flying*". Larger spores are more likely to be deposited on hosts [39–41]. Thus, even if smaller spores disperse farther than larger spores, the efficiency of dispersal in terms of probability of landing on a host would favor larger spores.

Interestingly, both spore production and size are strongly positively correlated in wild and agricultural habitats. Unlike plants and animals, the more spores produced, the bigger their size. This strong correlation probably reflects a linkage, even pleiotropy, between genes controlling spore production and spore size. Taking into account that larger spores maximize their ability to be deposited on canopy [40, 42], our results suggest that strains that produce more asexual spores have a better efficiency of dispersal. Why did selection not favor strains with higher spore production and larger spores? The variability for both spore size and production in our dataset probably account for some sort of trade-off as previously mentioned by Meerts [41] on Basidiomycota. Optimal spore size across species may vary according to life history. Thus, as *Venturia inaequalis* disperses asexual spores on leaves and fruits, mostly by splashing events over short distances [43–45], one might expect that larger spores should be favored in this species. We will discuss this point further when comparing strains from the wild and agricultural habitats.

Surprisingly, the germination rate of conidia did not correlate with any other trait. Larger spores were expected to have greater germination rates [17], but to our knowledge there is no clear demonstration of this correlation in fungi. Our results showed that spore size does not determine the ability to germinate. However, there is no data on survival rate of germinated spores. Indeed, observations showed that mortality could occur from several hours to days after germination. Such a correlation study between spore size and survival should deserve attention in the future.

### Traits related to the efficiency of pathogen’s dispersal were impacted during wild-to-crop transition

Our results demonstrated that apple domestication promoted modifications in traits related to dispersal of the apple scab fungus *Venturia inaequalis*. Among the four life history traits that were analyzed, both spore production and spore size showed significant differences between the two tested sampling sites. This means that agro-ecosystems might have selected a high production of larger spores for a more efficient short-distance dispersal. Indeed, wild apple trees, *M*. *sieversii*, in the Tien-Shan mountains of Kazakhstan make dense and large forests extending along valleys but also form patches in meadows at the peripheral of forests. Wild apple trees exhibit high phenotypic diversity and thus represent highly heterogeneous habitats for *Venturia inaequalis*. In contrast, *M*. *sieversii* trees around Almaty grow in a landscape dominated by agriculture, especially apple orchards that are characterized by a high homogeneity of genotypes and density. Higher spore size probably increases the probability to be deposited on neighbor apple trees in orchards. If a strain produces spores that are already adapted to the genotype of its host, likely this strain is also adapted to the hosts all around. Thus, selecting for high sporulation rate would also select for large spores, which potentially gives an advantage in competition in a homogeneous habitat. Conversely in wild heterogeneous habitats, a strain adapted to its host might not be adapted to the neighboring hosts. The potential for dispersal of relatively smaller spores might ensure reaching another canopy or distant compatible habitats [40, 41].

### Pestification by selection from standing phenotypic variation in the wild

The domestication process of plants and animals has often been associated to a strong disruptive selection favoring certain traits in each environment. In plants, transition between wild and cultivated forms was generally associated with different selection-targeted traits related to harvesting conditions, seed production or seedling competition [46].

We showed that phenotypic traits in agro-ecosystems were selected from standing variation in the wild. Indeed, rather than a strong disruptive selection, we observed that the range of each measured trait in the anthropized population mostly overlapped with the range of those traits in the wild population. We showed that phenotypic diversity within the domestic population preexisted in the wild population at least for the traits we have analyzed (Fig 3). The most trivial reason is that humans have not selected the pathogen for any agricultural or biotechnical trait of interest compared to plants or even domesticated fungi such as *Penicillium* [47] or *Saccharomyces* species [48]. As host tracking is a continuous process, we might suppose that plant modifications were progressive and therefore did not exert a strong selection on its pathogen. In the same line, it is noteworthy that *Malus x domestica* remains genetically very close to its wild relative species *M*. *sieversii*, *M*. *sylvestris* and *M*. *orientalis* [24]. We previously showed that moving this fungus outside its native range had not induced shifts in its reproductive mode, which is temperature dependent [22]. We also showed that limited gene flows occur between domestic and wild populations [29]. At this time, we cannot predict whether such low extent of genetic exchanges will maintain the observed phenotypic differences.

We presented here one of the first study of evolution of fitness-related traits in a pathogenic fungus triggered by the domestication of its host. As previously introduced by Saleh *et al*. [16], we called this process pestification. We found that this process led to an increase of the ability for efficient colonization of a homogeneous environment. In *Venturia inaequalis*, pestification mostly proceeded by selection from standing phenotypic variation in the wild. However, we do not know to which extent our conclusions can be transferred to other pathogens because evolution of fitness-related traits is highly dependent of the different life histories like predominance of sexual over asexual reproduction [17]. Our study also focused on conidiospores that ensure clonal dispersal. We have no information about phenotypic variation on ascospores. Dispersal of ascospores by air and rain splashes is crucial for the life cycle of *Venturia inaequalis* because it permits the very first colonization of buds and young leaves [43, 49, 50]. Nevertheless we found a high phenotypic diversity in the wild strains for the four traits we have analyzed.

We cannot exclude such a diversity for other traits like virulence or aggressiveness as suggested by Lê Van *et al*.[30]. This study emphasizes the potential threat for the durability of crop resistances that is represented by populations of pathogens living in wild environments [30, 51, 52].
